# Endocytic Pathways Unveil the Role of Syndecans in the Seeding and Spreading of Pathological Protein Aggregates: Insights into Neurodegenerative Disorders

**DOI:** 10.3390/ijms26094037

**Published:** 2025-04-24

**Authors:** Anett Hudák, Tamás Letoha

**Affiliations:** 1 Pharmacoidea Ltd., 6726 Szeged, Hungary; anett.hudak@pharmacoidea.eu; 2 Doctoral School of Theoretical Medicine, Albert Szent-Györgyi Medical School, University of Szeged, 6720 Szeged, Hungary

**Keywords:** endocytosis, heparan sulfate proteoglycans, syndecans, neurodegeneration, protein aggregation

## Abstract

Alzheimer’s disease and other neurodegenerative disorders are characterized by the accumulation of misfolded proteins, such as amyloid-beta, tau, and α-synuclein, which disrupt neuronal function and contribute to cognitive decline. Heparan sulfate proteoglycans, particularly syndecans, play a pivotal role in the seeding, aggregation, and spreading of toxic protein aggregates through endocytic pathways. Among these, syndecan-3 is particularly critical in regulating the internalization of misfolded proteins, facilitating their propagation in a prion-like manner. This review examines the mechanisms by which syndecans, especially SDC3, contribute to the seeding and spreading of pathological protein aggregates in neurodegenerative diseases. Understanding these endocytic pathways provides valuable insights into the potential of syndecans as biomarkers and therapeutic targets for early intervention in Alzheimer’s disease and other related neurodegenerative disorders.

## 1. Introduction

Neurodegenerative diseases represent a group of disorders characterized by the progressive degeneration of neurons, leading to irreversible cognitive and functional decline [1]. Alzheimer’s disease (AD) is the most prevalent of these disorders, affecting millions globally and being the leading cause of dementia [2,3,4]. Its hallmark features include the extracellular accumulation of amyloid-beta (Aβ) plaques and the intracellular formation of tau neurofibrillary tangles, both of which are believed to disrupt neuronal function and trigger neuroinflammation [4,5]. Other neurodegenerative diseases, such as Parkinson’s disease (PD), Huntington’s disease (HD), and amyotrophic lateral sclerosis (ALS), similarly involve the accumulation of misfolded proteins like α-synuclein and polyglutamine proteins [6,7,8]. These misfolded proteins form neurotoxic aggregates that contribute to neuronal dysfunction and cell death [9,10]. Despite the well-established role of protein aggregation in neurodegeneration, the exact mechanisms by which misfolded proteins accumulate and propagate—specifically, how they seed and spread—within the brain remain poorly understood [3,7,11]. This knowledge gap has limited the development of effective therapies and diagnostic tools for these diseases.

At the cellular level, the neuronal internalization of misfolded proteins plays a pivotal role in their aggregation and spreading [3,7,12]. Seeding refers to the process where small aggregates of misfolded proteins act as templates, inducing normally folded proteins to adopt a misfolded, pathogenic conformation, thus promoting the growth of larger aggregates. Spreading describes the ability of these aggregates to propagate from one cell to another, amplifying the toxic effects across the brain and contributing to the widespread pathology of neurodegenerative diseases. The process of endocytosis, by which cells engulf extracellular material, is central to the uptake of these proteins [3,7,13,14]. In neurodegenerative diseases, endocytic pathways are often dysregulated, resulting in enhanced uptake of misfolded proteins, their intracellular accumulation, and eventual neuronal damage [15,16]. Misfolded proteins such as Aβ, tau, and α-synuclein are internalized by neurons through specific endocytic routes, and their accumulation within endocytic compartments contributes to toxic fibril formation and synaptic dysfunction [3,7,16]. These proteins enter neurons via various endocytic mechanisms, including clathrin-mediated endocytosis, caveolae-dependent endocytosis, and lipid raft-mediated endocytosis, each of which is involved in the trafficking of cellular material to various intracellular compartments, such as early endosomes and lysosomes [3,13,16]. Upon internalization, misfolded protein aggregates traverse early endosomes, progressing to late endosomes and lysosomes [17,18]. In pathological conditions, the lysosomal degradation capacity is often impaired, leading to intracellular accumulation [18,19]. This impairment can result in the aggregation of toxic proteins being transferred intercellularly instead of being degraded, thus facilitating the spread of the aggregates and contributing to disease progression [3,7,20].

Importantly, syndecans (SDCs), a family of heparan sulfate proteoglycans (HSPGs), have emerged as critical regulators of endocytosis in neurodegenerative diseases [3,7,14]. SDCs are transmembrane proteins that mediate cellular interactions with extracellular matrix (ECM) components, growth factors, and various ligands [21,22]. They are distinguished by the presence of glycosaminoglycan (GAG) chains, particularly heparan sulfate (HS), which enables them to interact with a wide array of ligands, including misfolded proteins like Aβ, tau, and α-synuclein [3,7,23,24,25,26]. These interactions facilitate the internalization of toxic proteins through endocytic pathways, contributing to their aggregation and spread within neurons [3,7,16]. Among the four SDC family members—SDC1, SDC2, SDC3, and SDC4—SDC3 has garnered particular attention for its role in neurodegeneration [27]. SDC3 is highly expressed in neurons (hence, it is also referred to as N-SDC, where ’N’ stands for neuronal) and plays a role in the uptake of pathological proteins, promoting their internalization and enhancing fibrillation [3,7,14].

The cellular uptake of neurodegeneration-related misfolded proteins is complex and involves multiple mechanisms [16,21,28]. While clathrin-mediated endocytosis is a well-characterized pathway for the internalization of various cargo, recent studies suggest that lipid raft-dependent endocytosis is particularly important for the internalization of proteins like Aβ and tau [29,30,31]. Lipid rafts are specialized microdomains in the cell membrane enriched in cholesterol and sphingolipids, and they facilitate the recruitment of SDCs to the cell surface. The attachment of misfolded proteins triggers SDC oligomerization, facilitating the fibrillization and cellular internalization of misfolded proteins [3,7,32,33]. This pathway is thought to be crucial for the early stages of amyloid aggregation, where SDCs serve as seeding points and mediators of aggregate entry into the cell [3,7,34]. Once inside the cell, misfolded protein aggregates may accumulate within endocytic compartments, further enhancing their aggregation and triggering neurodegenerative processes [20,35,36,37,38].

Understanding the precise mechanisms by which SDCs facilitate the internalization and aggregation of misfolded proteins provides key insights into the pathophysiology of neurodegeneration [3,7]. The disruption of these endocytic pathways—either through mutations, dysregulation, or the overexpression of SDCs—can lead to an increased burden of toxic protein aggregates in neurons [3,7,39]. This may result in neuronal dysfunction, synaptic loss, and ultimately, neurodegeneration [40,41,42]. Importantly, the ability of SDCs to modulate endocytic pathways and influence protein aggregation makes them promising candidates for both diagnostic and therapeutic strategies [27,43,44]. By targeting SDCs or their associated endocytic pathways, it may be possible to limit the internalization and aggregation of misfolded proteins, offering a novel approach for slowing or halting the progression of neurodegenerative diseases [27].

This review aims to explore the role of endocytic pathways in neurodegenerative diseases, focusing on how these pathways reveal the hidden role of SDCs in the internalization and aggregation of misfolded proteins. We will discuss the molecular mechanisms of SDC-mediated endocytosis, their interactions with misfolded proteins, and how these processes contribute to the pathogenesis of neurodegenerative disorders such as AD. By understanding the endocytic mechanisms underlying the seeding and spreading of neurotoxic protein aggregates, we can identify potential biomarkers for early diagnosis and explore new therapeutic avenues to combat these devastating disorders [27].

## 2. Endocytic Pathways and Their Role in Neurodegeneration

Endocytosis is the cellular process by which cells internalize extracellular materials, including proteins, lipids, and other molecules [45,46]. This process plays a critical role in maintaining cellular homeostasis, regulating nutrient intake, and controlling the removal of waste products [45,47]. In neurons, endocytosis is essential for processes such as synaptic vesicle recycling, signal transduction, and membrane trafficking [45,48,49,50]. However, in neurodegenerative diseases, endocytic pathways become dysregulated, leading to abnormal aggregation, cellular internalization, and the spreading of aggregated misfolded proteins, such as Aβ, tau, and α-synuclein, which are key contributors to cellular toxicity and disease progression [3,7,16,35,51,52].

The various endocytic pathways, including clathrin-mediated endocytosis, caveolae-dependent endocytosis, and lipid raft-mediated endocytosis, play distinct roles in the internalization of extracellular material [32,53,54]. These pathways have been implicated in the uptake of misfolded proteins such Aβ, tau, and α-synuclein, which are central to the pathogenesis of neurodegenerative diseases like AD, PD, and other tauopathies and synucleinopathies [46,55,56,57,58].

SDCs, particularly SDC3, play a central role in endocytic pathways by facilitating the aggregation and uptake of misfolded proteins in neurons [3,7,27]. Understanding the molecular mechanisms of these endocytic pathways and the role of SDCs in regulating protein aggregation provides valuable insights into the pathogenesis of neurodegenerative diseases—including Parkinson’s disease, as both earlier and more recent studies have implicated SDC3 in its development, potentially opening new avenues for therapeutic intervention [59,60]. Although the role of SDC3 in ALS has not been extensively studied, recent work has begun to explore its involvement in different contexts of the disease, suggesting a broader relevance [61,62].

### 2.1. Clathrin-Mediated Endocytosis

**Figure 1:**
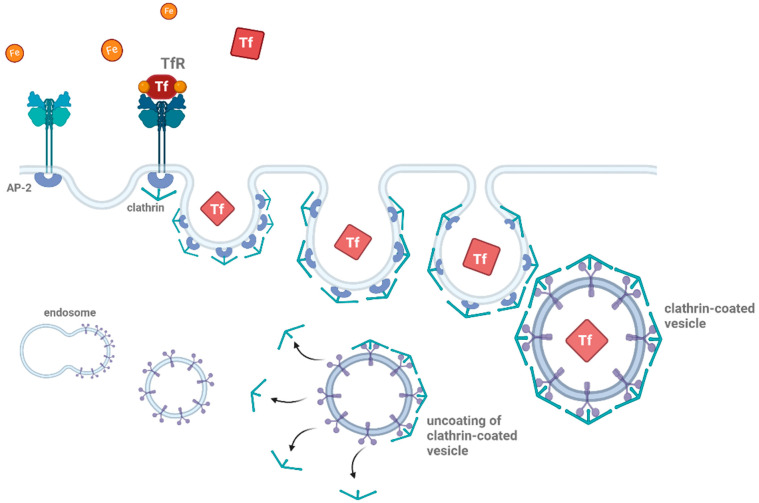
Schematic representation of clathrin-mediated endocytosis. Clathrin-mediated endocytosis begins with the binding of ligands (such as proteins or other molecules) to their receptors (e.g., transferrin, shown as TfR). The adaptor protein complex (AP-2) recruits clathrin to the site of receptor binding, forming a clathrin-coated pit. This pit then buds off from the plasma membrane, creating a vesicle encapsulating the receptor–ligand complex. The clathrin-coated vesicle undergoes uncoating, releasing the vesicle, which then fuses with early endosomes for further processing. This pathway is essential for cellular uptake and recycling of membrane proteins and ligands Thearrows indicate the uncoating process during clathrin-mediated endocytosis. The figure was created with BioRender.com (accessed on 11 March 2025).

Clathrin-mediated endocytosis, often referred to as ‘classical’ endocytosis, is the most well-studied form of endocytosis [63]. It involves the formation of clathrin-coated vesicles, which are responsible for the internalization of a wide range of extracellular material, including receptors, growth factors, and extracellular matrix proteins (Figure 1) [64,65]. The process begins with the recruitment of clathrin proteins to the plasma membrane, where they assemble into a lattice-like structure that buds off from the membrane, forming a vesicle [66,67]. This vesicle is then uncoated, and the internalized cargo is delivered to early endosomes for sorting and recycling [68].

In the context of neurodegenerative diseases, clathrin-mediated endocytosis is implicated in the internalization of misfolded proteins, such as Aβ and tau [3,7,56,69,70]. Evidence has shown that Aβ can be internalized by neurons through clathrin-coated pits, where it is subsequently delivered to intracellular compartments, where aggregation may occur [71,72]. Studies on misfolded proteins indicate that monomeric Aβ or other misfolded proteins preferentially enter cells via clathrin-mediated endocytosis [3,7]. Thus, clathrin-mediated endocytosis serves as the primary clearance mechanism for extracellularly accumulated protein monomers [73]. Disruptions in the regulation of clathrin-mediated endocytosis can thus lead to an accumulation of toxic protein aggregates, contributing to synaptic dysfunction and neuronal damage [74,75]. Furthermore, defective internalization or recycling of membrane proteins due to dysfunctional clathrin-mediated pathways can exacerbate cellular toxicity, playing a pivotal role in disease progression [13]. This highlights the importance of maintaining the proper function of ‘classical’ endocytic machinery in preventing the spread and accumulation of neurotoxic aggregates in neurodegenerative disorders.

### 2.2. Caveolae-Dependent Endocytosis

Caveolae are specialized lipid rafts rich in cholesterol and sphingolipids that form flask-like invaginations in the plasma membrane [76,77]. Caveolae-dependent endocytosis is a clathrin-independent process that involves the internalization of membrane-bound cargo via caveolae, which are stabilized by the caveolin family of proteins [78,79]. This pathway is important for the uptake of various molecules, including lipoproteins, growth factors, and toxins [80,81].

Recent studies suggest that caveolae-dependent endocytosis plays a significant role in the internalization and aggregation of Aβ and tau fibrils [82,83]. This pathway is thought to facilitate the selective uptake of misfolded proteins by neurons, contributing to their accumulation and aggregation within intracellular compartments [84]. Caveolin-1, a key protein in caveolae, has been shown to interact with Aβ, further supporting the idea that caveolae-mediated endocytosis is involved in the progression of AD [85,86]. Dysregulation of this pathway may exacerbate the accumulation of neurotoxic proteins and promote disease progression [87,88].

However, in studies with SDC-overexpressing cell lines lacking caveolin-1, Aβ aggregation and subsequent internalization occurred despite the absence of caveolin-1 [3]. These findings suggest that SDCs alone are sufficient to trigger intense fibrillation and internalization of misfolded protein aggregates, reducing the reliance on caveolae and related endocytic pathways in the seeding and spreading of neurodegeneration-associated protein aggregates.

### 2.3. Lipid Raft-Mediated Endocytosis

Lipid rafts are specialized microdomains within the cell membrane that are rich in cholesterol, sphingolipids, and certain proteins [89,90]. These rafts serve as platforms for the clustering of signaling molecules and the regulation of membrane trafficking [89,91,92]. Lipid raft-mediated endocytosis refers to the internalization of cargo via lipid rafts, which can occur through both clathrin-dependent and clathrin-independent mechanisms [53].

The lipid raft-mediated pathway plays a crucial role in the internalization of misfolded proteins, particularly Aβ and tau [93]. These proteins are thought to interact with lipid rafts, which facilitate their recruitment to the plasma membrane and subsequent internalization into neurons [94]. The lipid raft-mediated endocytosis of Aβ, for example, has been shown to be important for initiating fibrillation and promoting its aggregation [95]. Additionally, the internalization of Aβ via lipid rafts leads to the formation of toxic aggregates within endosomal compartments, contributing to neuronal toxicity and synaptic damage [96,97].

## 3. SDCs’ Structure, Function, and Endocytic Mechanisms

SDCs are a family of four transmembrane HSPGs that are expressed on the surface of a wide range of cell types, including neurons, where they play critical roles in cellular communication, signal transduction, and endocytosis [43,98,99]. These proteins are distinguished by their unique structure, which includes a core protein attached to one or more GAG chains, primarily HS, though some SDCs also contain chondroitin sulfate chains [3,7]. This structure allows SDCs to interact with a variety of extracellular ligands, including growth factors, extracellular matrix components, and neurodegeneration-related misfolded proteins, such as amyloid-beta (Aβ), tau, and α-synuclein [3,7,14,100].

SDCs, particularly SDC3, play a central role in the aggregation and cellular uptake of misfolded proteins in neurons [3,7,27]. Understanding the molecular mechanisms of these endocytic pathways and the role of SDCs in regulating protein aggregation provides valuable insights into the pathogenesis of neurodegenerative diseases and opens new avenues for therapeutic intervention [27,44].

### 3.1. SDCs’ Structure

**Figure 2:**
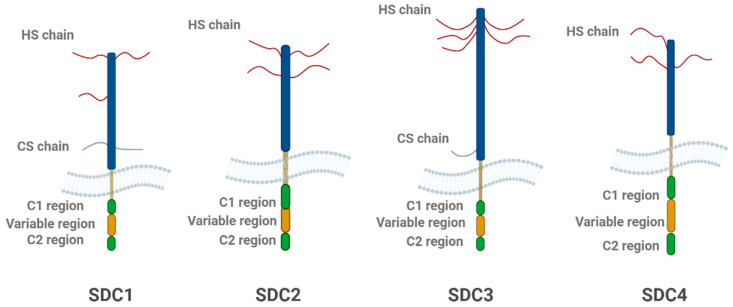
Structural schematic representation of the four SDC family members. Each SDC consists of a core protein with heparan sulfate (HS) chains and, in some cases, chondroitin sulfate (CS) chains. The extracellular domain includes the C1, variable, and C2 regions, which vary among SDC isoforms. SDC1 and SDC2 contain both HS and CS chains, while SDC3 and SDC4 primarily feature HS chains. The variable region is unique to each isoform, contributing to their functional diversity. The figure highlights the structural differences between the SDC family members, with SDC3 being particularly important in neurodegeneration. The figure was created with BioRender.com.

SDCs share a common structural organization, consisting of three distinct domains: the extracellular domain (ectodomain), the transmembrane domain (TMD), and the cytoplasmic domain (CD, see Figure 2) [101]. The ectodomain is the most variable across different SDCs and contains the GAG chains, primarily HS, though some isoforms also include chondroitin sulfate [102]. These GAG chains are crucial for SDCs’ ability to bind to a range of ligands [3,7,14]. The presence of these sulfated sugars allows SDCs to interact with high-affinity binding sites on ligand molecules, facilitating their internalization via endocytic pathways [28]. The TMD of SDCs is a single-pass region of 24–25 amino acids, which spans the lipid bilayer of the cell membrane [23]. This domain plays a key role in maintaining the structural integrity of the proteoglycan and is involved in interactions with intracellular signaling proteins [93]. Unique sequences in the transmembrane domain, such as alanine and glycine residues, promote protein dimerization, which is important for the formation of functional proteoglycan complexes [93,94].

### 3.2. SDCs’ Function

SDCs have diverse functions in cellular processes, with particular relevance to cell adhesion, migration, signal transduction, and endocytosis [103]. The GAG chains in the extracellular domain mediate interactions between SDCs and the extracellular matrix (ECM), facilitating cell–matrix adhesion [104]. These interactions are crucial for maintaining tissue architecture, neuronal growth, and migration during development [105,106]. Additionally, SDCs interact with various growth factors and cytokines, modulating their activity and contributing to processes such as wound healing, synaptic plasticity, and immune responses [107,108,109].

In neurons, SDCs, particularly SDC3, have been shown to regulate the formation and remodeling of synapses [110]. SDC3 is highly expressed in the brain, especially during early development, where it plays a key role in neuronal differentiation and axonal growth [111,112]. Furthermore, SDCs also participate in the regulation of signaling pathways that control neuronal survival, differentiation, and response to injury [98].

SDCs also play a critical role in regulating the internalization of extracellular material through endocytosis [3,7,14,113]. By mediating the uptake of growth factors, ECM components, and other ligands, they contribute to cellular homeostasis and signaling [114,115]. In neurodegenerative diseases, however, SDCs’ role in the internalization of misfolded proteins, such as Aβ, tau, and α-synuclein, has become a critical area of study [3,7,14,55].

### 3.3. SDCs’ Role in Endocytosis

SDCs are primarily involved in lipid raft-mediated endocytosis, enhancing the internalization of various ligands, including neurodegeneration-related misfolded proteins [3,7,16]. SDC-mediated endocytosis appears to occur independently of clathrin and caveolin, but in a lipid raft-dependent manner: ligands or specific antibodies induce clustering and redistribution of SDCs to lipid rafts, stimulating lipid raft-dependent, but clathrin- and caveolae-independent endocytosis of the SDC–ligand complex [3,7,116]. SDCS, particularly SDC3 and SDC4, play a crucial role in endocytosis through multiple mechanisms that may vary depending on the cell type and the specific cargo. Some studies suggest that SDCs, such as SDC4, actively bind to cargo and facilitate clathrin-mediated endocytosis, with clathrin recruitment being essential for vesicle formation [117]. In addition, SDCs do not merely internalize passively but may actively contribute to clathrin incorporation, thus mediating the endocytosis of various cargo molecules [117]. Other studies suggest that SDCs are not limited to clathrin-mediated endocytosis, as they also appear to modulate clathrin-independent pathways, highlighting their multifaceted role in various cellular processes [118,119]. In the context of neurodegenerative diseases, where the accumulation and internalization of toxic protein aggregates play a central role in disease progression, SDCs may serve as important modulators of these processes. Emerging evidence may specifically suggest that SDC3 predominantly mediates internalization through clathrin-independent endocytosis, for example, via flotillin-assisted vesicle formation [3,7]. However, it remains unclear whether SDC3 directly binds to cargo proteins and recruits to the clathrin machinery or acts more passively as a co-receptor modulating the activity of other endocytic regulators. SDCs have been shown to mediate the uptake of Aβ and tau fibrils, promoting their aggregation and accumulation within neurons [3,7,120]. The GAG chains of SDCs are crucial for their ability to bind these misfolded proteins, facilitating their entry into cells via lipid rafts [121]. Once internalized, the proteins may aggregate in endosomal compartments, forming toxic fibrils that disrupt cellular function [18].

Additionally, SDCs are involved in clathrin-mediated endocytosis, where they play a role in the uptake of various ligands, including extracellular matrix components and growth factors [117,122]. By influencing the regulation of clathrin-coated vesicle formation, SDCs can modulate the internalization of misfolded proteins and their subsequent aggregation [3,7,123]. The dysregulation of SDC expression or function can lead to an increased burden of toxic protein aggregates in neurons, contributing to neurodegeneration [3,7,27,124].

It is important to note that misfolded proteins exhibit distinct uptake mechanisms depending on their aggregation state [7,20]. When misfolded proteins such as Aβ, tau, and α-synuclein are in their monomeric form, they tend to be internalized via SDC-independent pathways, primarily through clathrin-mediated and caveolae-dependent endocytosis [3,7]. However, when these proteins aggregate into oligomers or fibrils, their uptake becomes SDC-dependent [3,7]. Aggregated misfolded proteins are preferentially internalized through SDC-mediated endocytosis, which relies on the interaction of the proteins with the HS chains of SDCs, particularly SDC3 [3,7,27]. It is still being investigated whether SDC3 directly induces conformational changes in Aβ, tau, or α-synuclein, or if it primarily functions as a carrier that facilitates the internalization and intercellular transfer of these aggregates. Current evidence suggests that SDC3 acts as a membrane-bound scaffold, presenting these aggregates for clathrin-independent endocytosis, although clathrin-mediated pathways may also play a role. This transition from SDC-independent to SDC-dependent uptake is crucial for the formation of toxic protein aggregates, which can accumulate in endocytic compartments, further enhancing their aggregation and contributing to neurodegeneration.

### 3.4. Lipid Raft-Dependent Endocytosis and Its Association with Neurodegeneration

SDCs’ role in protein internalization is closely linked to lipid raft-dependent endocytosis [3,7,109]. Enriched in cholesterol, sphingolipids, and signaling proteins, lipid rafts serve as platforms for the clustering of receptors, including SDCs, and are essential for the regulation of endocytic processes [124,125]. When misfolded proteins like Aβ and tau aggregate, they are preferentially internalized via lipid rafts, where they interact with SDCs [3,7]. The lipid raft-dependent endocytosis of Aβ and tau has been shown to be critical for initiating fibrillation and promoting their aggregation within neurons [3,7,15]. The lipid rafts not only facilitate the entry of these toxic proteins but also provide an environment for their further aggregation [83]. This aggregation within endosomal compartments leads to the formation of protein deposits that disrupt normal cellular functions and contribute to neuronal damage [126]. Studies have demonstrated that the disruption of lipid raft integrity or SDC function can impair the internalization of Aβ and tau, preventing the formation of toxic aggregates and reducing their neurotoxic effects [127,128]. This suggests that targeting lipid raft-mediated endocytosis or SDC function could provide therapeutic avenues for limiting the spread of pathological proteins and preventing the progression of neurodegenerative diseases.

## 4. SDCs in the Internalization of Pathological Protein Aggregates

The internalization of misfolded proteins such as Aβ, tau, and α-synuclein is a critical process in the pathogenesis of neurodegenerative diseases [3,7,59,125]. When these proteins aggregate, they have the potential to propagate disease by spreading throughout the brain, forming toxic fibrils and plaques that disrupt neuronal function [3,7,126]. SDCs, particularly SDC3, play a crucial role in the internalization and aggregation of these misfolded proteins, facilitating their entry into neurons and promoting their pathological accumulation [3,7,14,27,60]. Understanding the molecular mechanisms of SDC-mediated internalization and aggregation provides new insights into the pathogenesis of neurodegenerative diseases and identifies potential targets for therapeutic intervention.

### SDC3’s Role in Protein Fibrils’ Internalization and the Spread of Pathological Proteins

SDC3, a member of the SDC family primarily expressed in neural tissues, especially the brain, plays a critical role in neuronal development, synaptic function, and protein trafficking [27,109,127]. It is particularly important for mediating the internalization of misfolded proteins, such as Aβ and tau, which aggregate into fibrils or oligomers [3,7]. The HS chains on SDC3 act as high-affinity binding sites for these proteins, facilitating their recognition and uptake by neurons [3,7,128]. This interaction promotes the formation of toxic protein aggregates within neurons, impairing neuronal function, promoting synaptic loss, and triggering cell death [51]. SDC3’s ability to bind and internalize these aggregated forms of Aβ and tau contributes to the spread and accumulation of pathological proteins in neurodegenerative diseases [3,7].

The molecular mechanism behind SDC3’s interaction with misfolded proteins involves the sulfated HS chains on its extracellular domain, which bind to the charged regions of Aβ and tau [3,7,129]. When these proteins aggregate into oligomers and fibrils, they interact with SDC3, promoting their internalization into neurons [3,7]. Once inside, these aggregates are transported to early endosomal compartments, where they may fibrillate further, contributing to the formation of amyloid plaques and tau tangles, hallmark features of AD [130]. The internalization of these aggregated proteins is significantly enhanced, highlighting SDC3’s pivotal role in promoting the pathological aggregation that leads to cellular dysfunction [3,7,59].

**Figure 3:**
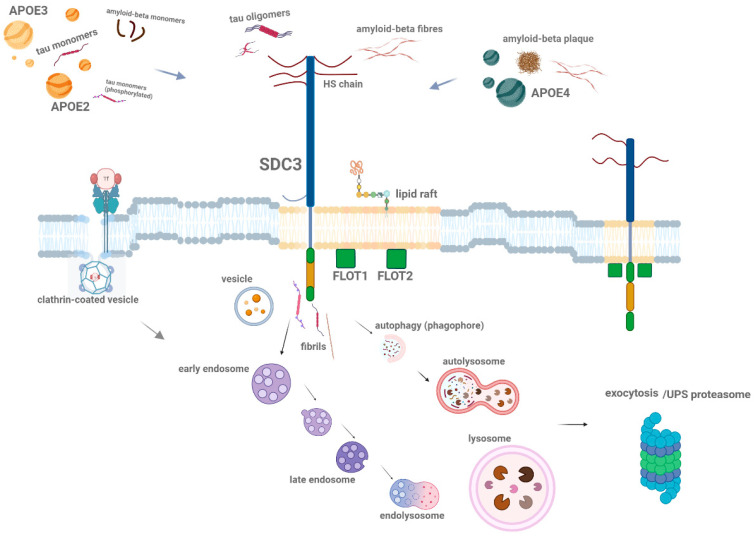
Schematic representation of SDC-mediated (highlighting the SDC3) internalization of misfolded proteins. This figure illustrates how different SDC family members on the cell surface interact with various misfolded protein aggregates. SDCs mediate the internalization of aggregated (polymeric) misfolded proteins, such as Aβ and tau fibrils, through lipid raft-dependent endocytic pathways. Additionally, the figure highlights the role of lipid raft-associated flotillins (FLOT1 and FLOT2) in the uptake of these aggregated proteins. Once internalized, the misfolded proteins are transported into endosomal and endolysosomal compartments, or autophagosomes, where they further aggregate and contribute to neuronal dysfunction. Alternatively, these proteins may either be secreted via exocytosis or degraded by the UPS (ubiquitin–proteasome system). The arrows indicate the direction of misfolded protein entry into the cell and their subsequent intracellular trafficking and processing pathways. The figure was created with BioRender.com.

SDCs, particularly SDC3, influence the spread of pathological proteins within the brain, enhancing their accumulation in neurons and promoting the formation of aggregates [3,7,14,27]. Once internalized, misfolded proteins like Aβ and tau may propagate their toxic effects by interacting with additional proteins, leading to the formation of fibrillar aggregates that contribute to plaque and tangle formation [106]. In neurodegenerative diseases, altered vesicular trafficking and impaired endocytic pathways, including lysosomal dysfunction, may influence the dynamics and localization of SDC3 [131]. While specific studies on SDC3 trafficking in neurodegeneration are limited, changes in endocytic machinery and protein aggregation could impact SDC3’s role in intercellular communication and the spread of toxic aggregates (Figure 3) Further research is needed to understand how neurodegeneration affects SDC3 trafficking and vesicle dynamics. This propagation process is central to the spread of neurodegenerative diseases, where localized aggregates can trigger the formation of new aggregates in neighboring cells, amplifying the pathological burden [131]. The spread of aggregated proteins, facilitated by SDC3, then affects synaptic communication and plasticity, further contributing to cognitive decline in neurodegenerative diseases.

The ability of SDCs to facilitate the seeding and spreading of pathological protein aggregates underscores their critical role in disease progression [3,7,27]. Studies have shown that the upregulation of SDCs, particularly SDC3, is associated with increased accumulation of misfolded proteins in both cultured neurons and animal models of neurodegeneration [3,7,14,27]. In these models, blocking SDC function or inhibiting lipid raft-mediated endocytosis reduces the internalization and aggregation of Aβ and tau, providing evidence that SDCs are key regulators of protein aggregation and toxicity [3,7,129].

By enhancing the internalization of misfolded proteins, SDCs facilitate their spread across neuronal networks, exacerbating the disease process [3,7,27]. This makes SDCs promising candidates for therapeutic intervention, as targeting their function could potentially halt the spread of toxic aggregates and slow or reverse the progression of neurodegenerative diseases. Additionally, developing targeted therapies to modulate SDC3 function could provide a novel strategy to mitigate the effects of pathological protein accumulation and delay the onset or progression of diseases like AD.

## 5. Apolipoprotein E and SDC Interactions in Neurodegeneration

**Figure 4:**
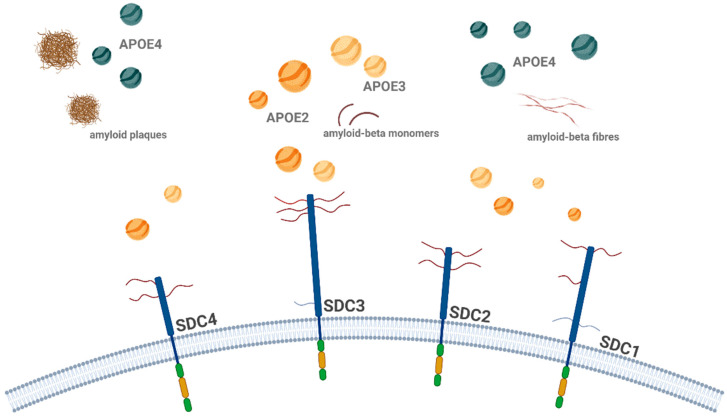
Schematic representation of ApoE isoform interactions with SDCs in Aβ aggregation and uptake. The figure illustrates the isoform-specific effects of ApoE on the cellular uptake and aggregation of Aβ, focusing on SDCs, particularly SDC3. ApoE2 and ApoE3 facilitate the uptake of ApoE, with ApoE2 enhancing the internalization of monomeric Aβ and reducing its extracellular aggregation, whereas ApoE4 has the opposite effect, promoting the accumulation of extracellular plaques. Once Aβ aggregates, ApoE2 inhibits the uptake of Aβ aggregates, while ApoE4 promotes it, leading to further aggregation. This figure highlights the complex interplay between ApoE isoforms and SDCs in Aβ pathology, with ApoE4 encouraging fibrillar Aβ aggregation and offering deeper insights into ApoE-dependent mechanisms in AD. The figure was created with BioRender.com.

Apolipoprotein E (ApoE) is a key protein in lipid metabolism, playing a crucial role in the transport and clearance of lipids and cholesterol [132,133]. In neurodegenerative diseases, particularly Alzheimer’s disease (AD), ApoE also influences protein aggregation and neuronal damage [14,134,135]. ApoE exists in three isoforms—ApoE2, ApoE3, and ApoE4—each of which affects the risk of developing AD [136,137]. ApoE4 is the most widely studied isoform and is associated with an increased risk of AD, while ApoE2 appears to have a protective effect [138]. ApoE interacts with a variety of cellular receptors, including HSPGs like SDCs, to modulate lipid and protein uptake, influencing the accumulation and aggregation of Aβ and tautwo hallmark proteins in AD (see Figure 4) [14]. Understanding the molecular basis of ApoE–SDC interactions provides new insights into the spread of pathological proteins and offers potential therapeutic strategies for targeting these interactions in the treatment of neurodegenerative diseases.

### 5.1. ApoE Isoforms and Their Role in AD

ApoE is essential for lipid transport within the brain, facilitating the movement of cholesterol and phospholipids to neurons, which is crucial for membrane integrity and synaptic function [139,140,141]. Beyond lipid metabolism, ApoE also influences the aggregation of misfolded proteins like Aβ and tau [14]. The three ApoE isoforms—ApoE2, ApoE3, and ApoE4—differ in their ability to interact with HSPGs on the surface of neurons and glial cells [142,143].

ApoE4, the most prevalent and risk-associated allele, has been shown to enhance the accumulation of Aβ in the brain, accelerating amyloid plaque formation [144]. In contrast, ApoE2 has a protective effect, promoting the clearance of Aβ and reducing the risk of AD [14,145]. The difference in the interaction between these isoforms and cellular receptors, including SDCs, may explain the varying effects of ApoE on the pathogenesis of AD [14].

### 5.2. Lipid Raft-Mediated Endocytosis of ApoE and Aβ

The interaction between ApoE and SDCs is closely tied to lipid raft-mediated endocytosis [14,146]. When ApoE interacts with Aβ, this complex is recruited to lipid rafts, where it is internalized into neurons through SDC-mediated endocytosis [14].

The lipid raft-dependent internalization of ApoE and Aβ is a critical step in the aggregation process [14,147]. Aggregated Aβ, when bound to ApoE, forms a complex that is preferentially internalized into neurons via lipid rafts [93]. Once internalized, Aβ aggregates are trafficked to endosomal compartments, where they may further aggregate, contributing to the formation of amyloid plaques [3,14]. The dysregulation of lipid raft-mediated endocytosis or altered ApoE isoform interactions with SDCs can exacerbate this process, leading to increased Aβ accumulation and neurodegeneration [14,148].

### 5.3. The Interaction Between ApoE and SDCs

SDCs are critical for the internalization of various ligands, including ApoE lipoproteins [14]. ApoE binds to SDCs’ HS chains, facilitating its internalization into neurons and glial cells [14,149]. The interaction between ApoE and SDCs has significant implications for the uptake and clearance of misfolded proteins like Aβ [14,150].

In AD, ApoE interacts with Aβ and enhances its deposition into plaques [14,147]. This interaction is isoform-dependent: ApoE4 binds to Aβ more strongly than ApoE2 or ApoE3, leading to increased Aβ accumulation in the brain [14,147]. This is thought to be due to ApoE4’s altered binding affinity for SDCs’ HS chains, which could facilitate the aggregation of Aβ and its deposition into plaques [14,151]. On the other hand, ApoE2’s weaker binding to Aβ and its increased ability to interact with HSPGs may promote the clearance of Aβ from the brain, suggesting a protective role in AD [14,151].

## 6. Endocytic Pathways as Therapeutic Targets in Neurodegeneration

The dysregulation of endocytic pathways is a central feature of neurodegenerative diseases, contributing to the aberrant internalization and accumulation of misfolded proteins such as Aβ, tau, and α-synuclein [3,7,14,152]. Understanding how these proteins are internalized into neurons, particularly through SDCs and lipid raft-mediated endocytosis, offers novel therapeutic opportunities. Targeting SDC-dependent endocytic pathways could provide a promising strategy to limit neurodegeneration, reduce the burden of protein aggregates, and improve outcomes in diseases like AD and PD.

A major challenge in developing therapeutic interventions targeting endocytic pathways is overcoming the blood–brain barrier (BBB), which selectively regulates the passage of molecules into the brain [153]. Effective therapies will need to bypass the BBB to modulate these pathways and address protein aggregation in neurodegenerative diseases [154]. Strategies targeting the mechanisms of protein aggregation, such as modulating endocytic pathways or regulating SDC function, have the potential to improve patient outcomes and provide new hope for treating AD and related disorders [27,134].

### 6.1. Modulating SDC Function to Prevent Protein Aggregation

As central mediators of lipid raft-dependent endocytosis, SDCs facilitate the fibrillation and entry of toxic aggregates into neurons [3,7,16]. Inhibiting SDC expression or blocking its interaction with misfolded proteins could reduce protein uptake and prevent their accumulation in neurons [3]. This approach to modulating SDC function presents an intriguing therapeutic strategy.

Several approaches could be considered for modulating SDC function. One potential strategy involves the development of small molecules or peptides that specifically inhibit the binding of misfolded proteins to SDC’s HS chains [155]. By preventing the interaction between SDCs and pathological proteins, this strategy could reduce the internalization of Aβ, tau, and α-synuclein, thereby limiting their aggregation and spread within the brain [3,7]. However, given the BBB’s restrictive nature, these peptides would need to be engineered to cross the blood–brain barrier effectively [156].

Alternatively, gene therapy approaches aimed at silencing SDC expression, particularly SDC3, which exhibits increased expression in AD brains, could reduce the internalization of these misfolded proteins and protect neurons from toxicity [157,158]. These gene therapy strategies face similar challenges in crossing the BBB, but advancements in targeted delivery systems, such as nanoparticle-based carriers or viral vectors, could help overcome this obstacle [159]. Although the SDC3 knockdown (KD) represents an attractive strategy to inhibit the SDC3-related seeding and spreading of pathological protein aggregates, it is important to emphasize that since SDC3 is a crucial modulator of synaptic plasticity and hippocampus-dependent memory, in vivo KD of SDC3 may lead to unintended side effects, particularly affecting learning and memory [110].

Another approach could involve enhancing the normal recycling and degradation pathways that SDCs regulate [160]. In their role in endocytosis, SDCs facilitate the trafficking of internalized proteins to endosomal and lysosomal compartments, where they can be degraded [161]. However, in the case of misfolded proteins, these degradation pathways are often impaired, leading to the accumulation of aggregates [3,7]. By improving the efficiency of protein degradation, it may be possible to reduce the accumulation of toxic aggregates and mitigate their damaging effects. Again, ensuring that these therapies can cross the BBB is a significant consideration.

### 6.2. Targeting Lipid Raft-Mediated Endocytosis

Lipid rafts play a critical role in the internalization of misfolded proteins [3,7,16]. Targeting lipid raft integrity presents a potential therapeutic strategy to inhibit the internalization of misfolded proteins and prevent their aggregation.

One promising approach involves the use of small molecules or cholesterol-lowering agents that disrupt lipid raft formation [162]. By inhibiting the recruitment of SDCs and other receptors to lipid rafts, it may be possible to reduce the internalization of misfolded proteins such as Aβ and tau, thereby limiting their pathological aggregation [44,163]. However, just as with targeting SDC function, delivering these therapies across the BBB remains a significant challenge.

Furthermore, targeting lipid raft-mediated signaling pathways that regulate protein internalization could provide an additional avenue for reducing protein accumulation and enhancing cellular clearance mechanisms. Similar to other strategies, overcoming the BBB is a critical consideration, as these therapies must be designed to cross the BBB efficiently and selectively target the brain.

### 6.3. ApoE and Therapeutic Modulation

ApoE is a key modulator of protein aggregation and internalization in neurodegenerative diseases [14,164]. As previously discussed, the ApoE4 isoform, which is associated with an increased risk of Alzheimer’s disease, enhances the internalization and deposition of Aβ in the brain [14,147]. By modulating ApoE4 function, it may be possible to shift the balance toward a more protective response. Another approach could involve enhancing the beneficial effects of the ApoE2 isoform, which is associated with the improved clearance of Aβ from the brain [145]. Gene therapies aimed at increasing the expression of ApoE2 or modulating its interactions with lipid rafts and SDCs could promote the clearance of Aβ and reduce the formation of amyloid plaques [165].

However, as with other therapeutic strategies, the BBB presents a significant challenge for delivering ApoE-modulating therapies to the brain [166]. Targeting ApoE–SDC interactions in the brain will require innovative methods to deliver these molecules across the BBB, ensuring that the therapies reach their intended targets without causing systemic side effects.

### 6.4. Exosome-Based Therapies for Protein Clearance

Exosomes are extracellular vesicles that play a role in intercellular communication and the transport of biomolecules, including proteins, lipids, and RNA [167]. Recently, exosome-based therapies have emerged as a potential strategy for enhancing the clearance of toxic proteins in neurodegenerative diseases [168]. Exosomes can be engineered to carry therapeutic agents, such as small molecules or RNA, that target misfolded proteins and facilitate their removal from the brain [169].

Exosomes also have the potential to be used to modulate the function of SDCs [170]. By designing exosomes that contain SDC-targeting peptides or antibodies, it may be possible to interfere with the internalization of misfolded proteins, limiting their aggregation [171]. Additionally, exosomes could be used to deliver gene therapy tools that promote the degradation of toxic protein aggregates or enhance cellular clearance pathways [172]. This approach represents a promising avenue for therapeutic intervention in diseases like AD and PD. Given their ability to cross the BBB, exosome-based therapies may offer a more effective method for delivering treatments directly to the brain [173].

## 7. Conclusions

Neurodegenerative diseases, including AD, PD, and other tauopathies and synucleinopathies, are characterized by the accumulation and aggregation of misfolded proteins, which disrupt neuronal function and lead to cognitive decline, synaptic loss, and cell death [3,7,174]. Despite significant advances in our understanding of these diseases, effective treatments remain scarce. The study of endocytic pathways and the role of SDCs in the internalization and aggregation of these misfolded proteins provides critical insights into disease progression and highlights novel therapeutic targets.

SDCs, particularly SDC3, are central mediators in the uptake of misfolded proteins such as Aβ, tau, and α-synuclein [3,7,14,27]. These transmembrane HSPGs play a critical role in the aggregation and spread of pathological proteins in the brain [3,7]. Through lipid raft-dependent endocytosis, SDCs facilitate the aggregation and internalization of these proteins, promoting their entry into neurons [3,7,14]. Endocytosis is the process by which cells take up molecules and particles from their environment by enclosing them in vesicles [45]. Unlike classical endocytic pathways, such as clathrin-mediated endocytosis, which involves clathrin-coated pits and adaptor proteins, or caveolin-mediated endocytosis, which utilizes caveolae and caveolin proteins within lipid rafts, SDC-mediated internalization operates via distinct mechanisms [78,175]. SDCs, particularly SDC3, act as cell surface proteoglycans that bind ligands through their heparan sulfate chains and facilitate internalization through lipid raft-associated or clathrin-independent routes [7]. While clathrin pathways are known for internalizing nutrients and receptors (e.g., transferrin, LDL) and caveolin is involved in cholesterol regulation and mechanosensing, SDCs predominantly handle extracellular matrix components, growth factors, and cell adhesion molecules [176,177,178].

In contrast, exocytosis is the process by which cells excrete substances through vesicles that fuse with the plasma membrane [176]. These complementary mechanisms regulate cell motility, with endocytosis mediating the uptake and potential intracellular spread of pathogenic proteins, while exocytosis may contribute to the release and extracellular spread of toxic aggregates [177]. This process contributes to the formation of toxic protein fibrils and plaques, which are hallmark features of neurodegenerative diseases. Additionally, SDCs influence the propagation of these toxic aggregates across neuronal networks, amplifying their pathological effects and accelerating disease progression [3,7]. While some studies have demonstrated a regenerative and protective role for SDC3 in vivo, conflicting reports suggest that SDC3 may also promote pathological aggregation in other neurodegenerative conditions [3,7,59,123]. These inconsistencies may be attributed to disease stage-specific or cell type-dependent effects, underscoring the need for further studies comparing the relationship between acute injury models and chronic neurodegenerative diseases. The dual role of SDC3 appears to vary depending on disease progression and cellular environment [178,179]. In the early stages of Alzheimer’s disease, SDC3 expression may have neuroprotective effects through the pleiotropin signaling pathway [180]. However, as the disease progresses, persistent SDC3 expression may promote Aβ accumulation and plaque formation [3,7]. In addition, the effect of SDC3 varies by cell type; in neurons, it may promote Aβ uptake, while in endothelial cells, it may influence inflammatory responses and monocyte migration [27].

Targeting the molecular mechanisms underlying SDC-mediated endocytosis and the aggregation of misfolded proteins offers exciting new opportunities for therapeutic intervention. Strategies to modulate SDC function, disrupt lipid raft integrity, or target specific ApoE–SDC interactions could reduce the internalization and aggregation of toxic proteins, potentially slowing or halting the progression of neurodegenerative diseases [14,181]. Additionally, exosome-based therapies, which could be engineered to carry therapeutic molecules, hold promise for enhancing protein clearance and modulating endocytic processes, offering a potential approach to restoring protein homeostasis in the brain [182].

In addition to synthetic approaches, emerging studies suggest that certain natural compounds, such as dermatan sulfate analogs, may modulate SDC3 function and its interactions with pathological protein aggregates [151]. These compounds offer a promising, potentially less toxic alternative by mimicking or interfering with endogenous GAG-mediated binding events, thus altering misfolded proteins’ internalization or aggregation capacity [151]. Further investigation is needed to determine their efficacy and bioavailability in vivo, particularly in the context of blood–brain barrier permeability and long-term safety.

However, one of the major challenges in developing these therapies is overcoming the BBB [27,183]. The BBB restricts the delivery of many therapeutic agents to the brain, making it difficult to target endocytic pathways and protein aggregation effectively [184]. Innovative drug delivery systems, such as nanoparticles, viral vectors, and exosome-based carriers, are emerging as potential solutions for crossing the BBB and delivering treatments directly to the brain [185].

The study of endocytic pathways and SDCs in neurodegenerative diseases is still in its early stages, but it holds immense promise for the development of novel therapeutic strategies. By further elucidating the role of SDCs in the internalization and aggregation of misfolded proteins, we can gain a deeper understanding of the pathogenesis of neurodegenerative diseases and identify new avenues for treatment. Ultimately, targeting these pathways could offer hope for millions of individuals affected by Alzheimer’s, Parkinson’s, and other neurodegenerative disorders, improving their quality of life and slowing the progression of these debilitating diseases.
